# Exploring the Ligand Binding and Conformational Dynamics of the Substrate-Binding Domain 1 of the ABC Transporter GlnPQ

**DOI:** 10.1021/acs.jpcb.4c02662

**Published:** 2024-08-02

**Authors:** Mariia Nemchinova, Gea K. Schuurman-Wolters, Jacob J. Whittaker, Valentina Arkhipova, Siewert J. Marrink, Bert Poolman, Albert Guskov

**Affiliations:** † Groningen Institute for Biomolecular Sciences and Biotechnology, University of Groningen, 9747AG Groningen, The Netherlands

## Abstract

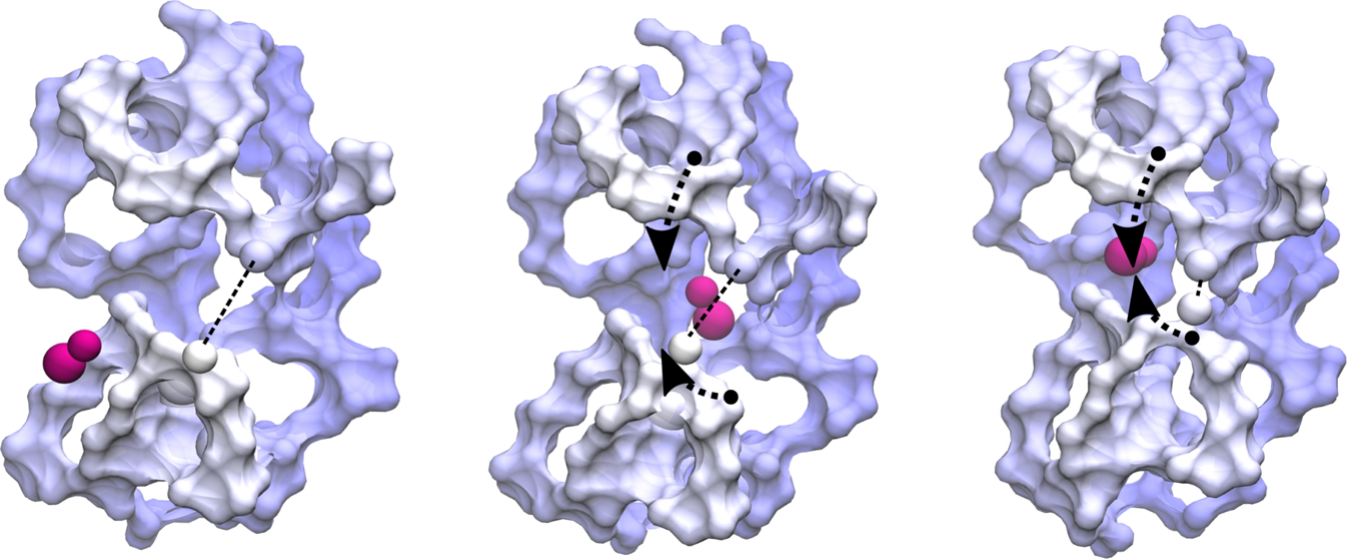

The adenosine triphosphate (ATP)-binding cassette (ABC) importer GlnPQ from *Lactococcus lactis* has two sequential covalently linked substrate-binding domains (SBDs), which capture the substrates and deliver them to the translocon. The two SBDs differ in their ligand specificities, binding affinities and the distance to the transmembrane domain; interestingly, both SBDs can bind their ligands simultaneously without affecting each other. In this work, we studied the binding of ligands to both SBDs using X-ray crystallography and molecular dynamics simulations. We report three high-resolution structures of SBD1, namely, the wild-type SBD1 with bound asparagine or arginine, and E184D SBD1 with glutamine bound. Molecular dynamics (MD) simulations provide a detailed insight into the dynamics associated with open-closed transitions of the SBDs.

## Introduction

Adenosine triphosphate (ATP)-binding cassette (ABC) transporters represent a major superfamily of transmembrane proteins, universally distributed among all organisms, and play an important role in a variety of cellular processes.^1−5^ ABC transporters are implicated in the transport of very diverse molecules, such as nutrients, metabolic products, and drugs,^6−10^ antibiotic and drug-resistance by exporting certain toxic substances,^11−13^ biogenesis of extracellular complex polysaccharides,^14^ lipid trafficking,^14−16^ cell volume regulation,^17,18^ and also in large-scale accumulation of signaling molecules for intercellular communication.^19−21^ The importance of ABC proteins and the diversity of their physiological roles are also of biomedical and clinical relevance due to their links with genetic diseases or the consequences of their dysfunction.^4^

All canonical ABC transporters consist of two transmembrane domains (TMDs) and two highly conserved cytoplasmic nucleotide-binding domains (NBDs),^22^ which power transport through the hydrolysis of ATP.^1,6,23^ In addition, importers possess either soluble binding proteins (SBPs) or tethered substrate-binding domains (SBDs) that mediate the initial binding of the substrate and delivery to the translocation subunit.

**Figure 1:**
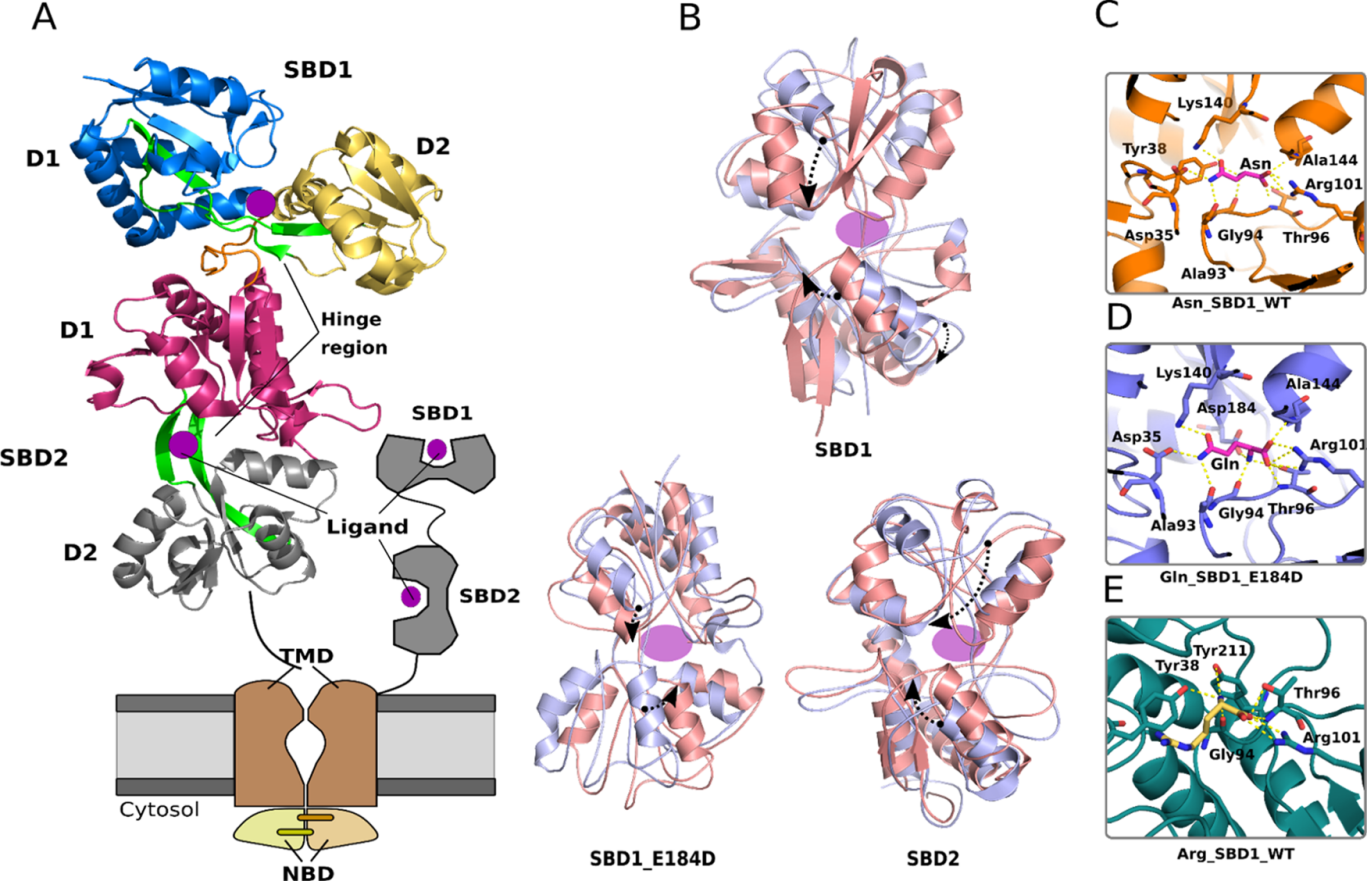
Overview of the structure of the SBD domains of GlnPQ. (A) Crystallographic structure of the tandem SBD1–2 (shown as a cartoon, PDB ID: 6H30) and schematic representation of the GlnPQ transporter. The homodimer is composed of two subunits: GlnP comprising the TMD linked to SBD2 and SBD1 and GlnQ (NBDs). The large (D1) and small (D2) subdomains of SBD1 and SBD2 domains in blue, dark magenta, yellow, and gray, respectively. The hinge region of both domains is in green. Ligands are depicted in magenta. The linker region is close to the hinge region of SBD1 in orange. (B) Superposition of simulated liganded (pink) and unliganded (light purple) structures to show the difference in the Venus-fly trap movement of the SBDs upon binding of a substrate (magenta) during coarse-grained (CG) molecular dynamics (MD) simulations. Sequences were aligned using the hinge region as an anchor point. The corresponding motions are highlighted with black arrows. Compared to the SBD1 wild type (SBD1, top), the E184D mutant adopts a semiopen state (SBD1 E184D). (C–E) Close-up view of binding site in crystal structures of SBD1 wild type (C) (Asn SBD1 WT, top) and SBD1 E184D mutant (D) (Gln SBD1 E184D, center) in the presence of the arginine and glutamine (shown in violet stick representation), respectively. Arginine binding (in yellow) to the open structure of SBD1 (E) (Arg SBD1 WT, bottom). Zoom-in on hydrogen bonding interaction between arginine and residues in the binding site of SBD1. Residues interacting with glutamine are labeled by number. The dashed lines show polar contacts in the binding sites, which range from 1.8 to 3.6 Å.

GlnPQ is an ABC transporter involved in the uptake of glutamine, glutamic acid, and asparagine^24,25^ and was found to be a robust model protein to study the mechanism of substrate delivery from SBDs to the translocon.^1,6,23^ In GlnPQ, there are two sequentially bound SBDs, where SBD2 is proximal to the translocator and SBD1 is distal.^26^ SBDs operate in a Venus-fly trap mechanism,^4,17,27,28^ where the binding site for a substrate is located between two lobes of an SBD (Figure 1), which is connected by two antiparallel β-strands (the hinge region).^29^ SBDs convert from open ligand unbound to the closed ligand-bound state, and the dwell time of the latter can be a rate-determining step in the transport cycle.^24^

Previously, we conducted isothermal titration calorimetry (ITC) experiments, which revealed that SBD1 has a relatively high affinity for asparagine (Table S1 summarizes the binding characteristics of SBDs), with an equilibrium dissociation constant (*K*_D_) = 0.2 μM and a significantly lower binding affinity for glutamine (*K*_D_ = 92 μM).^25^ Intriguingly, the formation of the SBD1–2 tandem has no significant effect on Asn/Gln binding to SBD1 in tandem (*K*_D_ of 0.4 ± 0.1 and 180 ± 100 μM, respectively).^26^ The solitary SBD2 binds glutamine with a *K*_D_ of 0.9 ± 0.2 μM,^26^ which is also similar to the one in tandem (*K*_D_ = 0.6 ± 0.2 μM).^26^ In addition, it has been shown through uptake experiments that glutamate is transported by both SBD1 and SBD2 domains.^30−32^ The affinity can be easily tweaked by mutagenesis, for example, the double E184D_V185E and the single E184D mutation in the SBD1 domain yield a significantly increased affinity for glutamine (*K*_D_ of 1.3 ± 0.15 μM and 1.0 ± 0.1 μM, respectively)^24^ (Table S1). Furthermore, no ligand transport is observed in SBD1(E184W) and SBD2(D417F) mutants due to their inability to form a closed state.^24,33^

To date, a number of crystallographic structures and some structural information from NMR on individual SBD1 and SBD2 domains are available.^25,29^

The high-resolution crystal structures of SBD1 and SBD2 were obtained in unbound (*apo* state) open conformation (1.4 Å (PDB ID: 4LA9) and 1.5 Å (PDB ID: 4KPT) resolution, respectively) and a closed conformation of a glutamine-bound SBD2 (0.95 Å) resolution (PDB ID: 4KQP).^25,29^

The crystal structure of the SBD1–2 tandem is also reported, albeit with much lower resolution of 2.8 Å.^26^ Notwithstanding the divergence of the sequence by more than 50%, the overall fold of the domains and binding pockets in both SBDs are very similar.^24^ Each SBD consists of two α/β subdomains, where the large α-subdomain (D1) is arranged by 29–113, 207–251 and 255–345, 444–484 residues in SBD1 and SBD2, respectively, and the small β-subdomain (D2) is formed by 114–206 residues in SBD1 and residues 346–438 in SBD2.^26,34^ The substrate-binding site is localized between the subdomains in both SBDs (see Figure 1A for the nomenclature).

However, crystallographic “snapshot” structures are insufficient to understand the dynamics of binding and conformational changes of SBDs. Targeted global structural rearrangements have been previously investigated using single-molecule spectroscopy and single-molecule Förster resonance energy transfer (smFRET) experiments on single SBD1 and SBD2. It has been shown that substrate binding is linked to a conformational change of the corresponding SBD from an *apo* (ligand unbound) to a closed (ligand bound) *holo* form.^24,26,27,33,35^ In the presence of the ligand, the closed state of the SBDs was observed more frequently, and there was no effect on the lifetime of the closed-liganded state; it was approximately equal to the ligand-free closed state.^26^ The binding of substrate leads to the movement of the subdomains relative to each other, as shown in Figure 1B. This movement can be described as an induced-fit-type ligand-binding mechanism.^24,35^ Additionally, it has been shown that the closed conformation triggers ATP-hydrolysis and transport.^24^ By comparison of the FRET efficiency histograms of the different ligand complexes, it was noted that some SBDs have a variety of ligand-bound active conformations, and in this case, selectivity exists due to the rate of domain opening or the selectivity provided by the translocator.^35^ Despite available structural and experimental data on the ligand-binding and global conformational changes of GlnPQ SBDs 1 and 2, a comprehensive model of the molecular mechanism of substrate binding to SBDs and associated conformational changes in tandem is still missing. Moreover, without the E184D SBD1 structure, it is hard to understand the impact of the E184D mutation on the binding affinity as previously observed experimentally.^25,26^ To resolve that, we have determined high-resolution X-ray structures of wild-type SBD1 in the presence of Asn and SBD1 soaked with Arg, and the E184D SBD1 mutant in the presence of Gln. Prompted by these findings, we performed coarse-grained (CG) molecular dynamics (MD) simulations to achieve more detailed insight into the mechanism of ligand binding.

## Methods

### Transformation and Cloning

The genes for SBD1 of GlnPQ were amplified by polymerase chain reaction (PCR) using genomic DNA of *Lactococcus lactis* IL1403 and nLIC complementary primers.^36^ The resulting PCR product was treated with T4 DNA polymerase and inserted into the SwaI site of the T4 polymerase-treated pBADnLIC vector. The resulting plasmids were transformed to *Escherichia coli* MC1061 and grown in Luria broth (LB). Ampicillin (Invitrogen) with 100 μg/mL concentrations was used as a selection marker. Site-directed mutagenesis was performed on the pBADnLIC plasmids by two different methods: (1) using primers with silent mutations to add a restriction site and the PCR product amplified using Phusion polymerase (Fermentas) and (2) via the USER cloning method,^37^ where uracil is introduced in the primers to create overhangs and amplification is done using PfuX7 polymerase. The resulting plasmids were transformed to the GlnPQ null strain, *L. lactis* GKW9000, and cells were grown on M17 broth supplemented with 1% (w/v) glucose plus 5 μg/mL chloramphenicol.

### Protein Expression and Purification

A single colony of *E. coli* MC1061, overexpressing wild-type or mutant variants of the soluble SBDs, was cultivated aerobically in LB supplemented with 100 μg/mL at 37 °C. Expression was induced at an OD_600_ of ∼ 0.5 by adding 2 × 10^–4^% arabinose and growing of the cells for another 2 h. The cells were isolated by centrifugation (4500*g*, 15 min, 4 °C), washed in 100 mM potassium phosphate (KPi) pH 7.5, and resuspended in Buffer A (50 mM KPi, pH 7.5, 20% (v/v) glycerol). Furthermore, the cells were broken with a Maximator High Pressure Homogenizer Type HPL6 (Maximator GmbH). The cells were supplied with a 10 μg/mL of DNase, 1 mM MgCl_2_, and 1 mM phenylmethanesulfonyl fluoride (PMSF) and broken in a single passage (25 kPsi, 4 °C). Afterward, the cell debris was removed by ultracentrifugation (150,000*g*, 90 min, 4 °C), and the resulting cell lysate was flash frozen in liquid nitrogen and stored at −80 °C in aliquots of 5 mL. To purify the wild-type or mutant proteins, an aliquot of the cell lysate was thawed and mixed with 3.5 mL Ni^2+^-Sepharose resin in Buffer B (50 mM KPi pH 8.0, 200 mM KCl, 20% (v/v) glycerol, 20 mM imidazole). The supernatant was incubated for 1h at 4 °C while gently rocking with Ni^2+^-Sepharose resin. Subsequently, the mixture was poured into a column (BioRad), the unbound material was allowed to flow through, and the column was washed with 20 times CV of Buffer B, supplemented with 50 mM imidazole. Protein was eluted with 500 mM imidazole in buffer C (50 mM KPi pH 8.0, 200 mM KCl, 20% (v/v) glycerol, 500 mM imidazole). Afterward, the purified protein was combined with tobacco etch virus (TEV) protease at a 1:40 w/w ratio and subjected to overnight dialysis against Buffer D (50 mM Tris-HCl pH 8.0, 0.5 mM EDTA plus 0.5 mM DTT). To purify the wild-type or mutant proteins, an aliquot of the cell lysate was thawed and mixed with 3.5 mL Ni^2+^-Sepharose resin in Buffer B (50 mM KPi pH 8.0, 200 mM KCl, 20% (v/v) glycerol, 20 mM imidazole). The supernatant was incubated for 1h at 4 °C while gently rocking with Ni^2+^-Sepharose resin. Subsequently, the mixture was poured into a column (BioRad), the unbound material was let to flow through, and the column was washed with 20 times CV of Buffer B, supplemented with 50 mM imidazole. Protein was eluted with 500 mM imidazole in buffer C (50 mM KPi at pH 8.0, 200 mM KCl, 20% (v/v) glycerol, 500 mM imidazole). Afterward, the purified protein was combined with TEV protease at a 1:40 w/w ratio and subjected to overnight dialysis against Buffer D (50 mM Tris-HCl pH 8.0, 0.5 mM EDTA, and 0.5 mM DTT). Elution fractions were applied to the second Ni^2+^-sepharose column (1 mL) that preliminarily was equilibrated with Buffer E (50 mM KPi, pH 8.0 and 200 mM KCl), then the flow through was collected, and the column was washed with 2× CV of Buffer E. Protein-containing fractions were stored in 1 mL aliquots at a concentration between 1 and 3 mg/mL in −80 °C after flash freezing in liquid nitrogen. Prior to further experiments, the eluted protein was thawed and purified by size exclusion chromatography on the Superdex 200 (GE Healthcare) in the gel filtration Buffer F (20 mM Hepes-NaOH, pH 7.5, and 150 mM NaCl).

### Crystallization, Data Collection, and Structure Determination

Crystals of SBD1 and mutant were obtained with the vapor diffusion technique (hanging drop) in a 1:1 v/v ratio with the following condition: 0.1 M MES pH 6.0, 5% PEG3000, and 30, 35, or 40% PEG400. To obtain MES-free crystals, reservoir solution was exchanged to 40% PEG 600, NaH_2_PO_4_/citric acid, pH 4.2. Crystals soaked with arginine were prepared from MES-free crystals by soaking them in a reservoir buffer containing 10 mM arginine. Prior to the crystallization of SBD1 with asparagine, protein was concentrated to 20–30 mg/mL, and 5 mM substrate was added. The composition of the reservoir solution was 0.1 M NaAc, pH 4.5, 0.05 M CaAc plus 40% v/v propanediol. Liganded SBD1(E184D) (concentrated to 23 mg/mL) crystals were grown in the presence of 1 mM l-glutamine. The reservoir solution consisted of 70% (v/v) MPD and 0.1 M Hepes, pH 7.5. Crystals were flash frozen in liquid nitrogen and brought to the synchrotron for analysis. Data sets were collected at beamline X06DA (SLS, Villigen) and beamline ID23-1 (ESRF, Grenoble).

Data sets were processed with XDS,^38^ and the structures were solved by Molecular Replacement with Phaser 2.1.4 of the CCP4 program suite^39^ using the previously published model of SBD1 (PDB ID 4KPT). Manual rebuilding was done with COOT^40^ and refinement with Phenix refine.^41^ Refined models were deposited in the PDB repository. Data collection and refinement statistics are summarized in Table 1.

**Table 1:** Data Collection and Refinement Statistics

	SBD1-Asn	SBD1(E184D)-Gln	SBD1-Arg
PDB ID	6FXG	8B5D	8B5E
wavelength	0.916	0.916	0.916
resolution range	44.2–1.7 (1.8–1.7)	47.77–2.0 (2.07–2.0)	38.66–1.6 (1.66–1.6)
space group	*P*2_1_	*P*2_1_	*P*1
*a*, *b*, *c* (Å)	41.87, 74.27, 110.24	42.61, 91.50, 57.91	34.67, 53.08, 54.08
α, β, γ (deg)	90.00, 90.55, 90.00	90.00, 107.81, 90.00	92.40, 92.37, 94.11
unique reflections	135138	27548	49268
completeness (%)	97.6 (95.6)	96.27 (96.63)	97.20 (95.88)
mean *I*/sigma (*I*)	15.2 (2.1)	8.8 (2.0)	12.1 (2.4)
*R*-meas	4.0 (10.1)	5.2 (25.8)	5.1 (22.5)
*R*-work (%)	13.6	19.88	14.69
*R*-free (%)	17.0	26.46	21.81
protein atoms	10455	3449	3589
ligand atoms	45	43	124
solvent atoms	952	215	266
RMS (bonds) (Å)	0.009	0.009	0.010
RMS (angles) (deg)	0.981	1.14	1.56
Ramachandran favored (%)	98.36	96.61	97.53
Ramachandran allowed (%)	1.64	3.16	2.02
Ramachandran outliers (%)	0	0.23	0.45
rotamer outliers (%)	0	0.83	2.62
clashscore	2.0	3.60	5.37
average B-factor	23	40.40	26.89
macromolecules	25.1	40.09	25.73
ligands	13	56.31	51.01
solvent	30.1	42.22	37.74

### Molecular Dynamics Simulations

#### CG Simulations

Coarse-grained (CG) MD simulations were performed with the Martini 3^42,43^ force field in combination with a Go̅-like model,^44^ using the Gromacs software (version 2020).^44−46^ The starting structure for the CG MD simulations is the obtained high-resolution crystallographic structure of SBD1 wild-type (WT) in the presence of l-Arg (PDB ID 8B5E), where the ligand was removed from the binding site. We also performed CG MD simulations of the E184D SBD1 mutant based on the obtained X-ray structure (PDB ID 8B5D) and the SBD1–SBD2 tandem of GlnPQ (PDB ID 6H30), as well as the isolated SBD2 domain of the tandem. To exclude the unlikely possibility of overfitting during refinement, the files were checked with the PDB_REDO^47^ Web server. Systems containing the single SBDs were solvated by ∼28080 CG water beads (representing 112,320 water molecules) in a cubic box (15.0 nm × 15.0 nm × 15.0 nm) and neutralized, and 0.15 M NaCl was added. In the case of the tandem of GlnPQ, the systems were solvated with ∼66290 CG water beads (265160 water molecules), resulting in a box of 20.0 × 20.0 × 20.0 nm. Again, 0.15 M NaCl was added to the system after the neutralization. For each system simulated, a single copy of a ligand (Asn, Gln, Arg, and His) was added randomly. In Martini 3, Asn is represented as a pair of P2-SP5 beads, Gln as a pair of P2–P5 beads, Arg as a triplet of P2-SC3-SQ3p beads, and His as a P2-TC4-TN6d-TN5a construct, all beads connected via harmonic bonds. The P2 bead in all cases represents the amino acid backbone. Despite the limited resolution in Martini, similar amino acids, such as Asn and Gln, can still be meaningfully distinguished. The P5 bead represents very polar fragments such as acetamide and propanamide (the side chain analogues of Asn and Gln). The larger size of the Gln residue compared to Asn is accounted for by having a regular bead type for the latter and a smaller bead type (prefix S) for the former. This choice of bead type has been validated previously by computing partitioning free energies of the side chain analogues between different organic solvents, reproducing the more hydrophilic nature of Asn versus Gln. The equilibrium bond length has been optimized to reproduce the solvent-accessible surface (SASA) of the amino acids with respect to all-atom models, a standard procedure in Martini 3. Together, the differences in size and polarity allow us to discriminate between these amino acids at the Martini level of resolution. More details about the amino acid force field are given in ref (67). For all systems in the presence of the ligand, the movements of the ligand were limited to reside within the selected distance from the protein (20 Å), using a flat-bottom potential. The use of a restraining potential keeps the ligand in the vicinity of the binding site, avoiding sampling ligands diffusing in the surrounding aqueous environment. Note that the movement of the ligands in and out of the pocket is unrestrained. The WT SBD1 domain was also simulated in the absence of a ligand.

The CG structures of the proteins were generated using the program *martinize2.py* (see https://github.com/marrink-lab/vermouth-martinize).^48^ To stabilize the protein’s secondary and tertiary structure, we created a network of Go potentials using the GoMARTINI tool with default settings.^44^ The variant of the OV (the overlap of enhanced the van der Waals radii spheres) + rCSU (a variant of the contact of structure units) contact map, which takes into account the chemical properties of the atoms in contact, was used.^49^ The contact maps were obtained from http://info.ifpan.edu.pl/∼kwolek/rcsu/http://info.ifpan.edu.pl/∼kwolek/rcsu/ (with radii used by Tsai et al.^50^ and Fibonacci number equal 17), where the definition of contacts in the rCSU algorithm and a server to run various examples can be found.

To integrate the equations of motion, the leapfrog propagator was employed in combination with the Verlet cutoff scheme and a buffer tolerance of 0.005 kJ/mol. van der Waals interactions were treated using the cutoff scheme with a cutoff of 1.1 nm.^51^ The temperature and pressure were controlled with a velocity-rescale thermostat (reference temperature *T* = 303.15 K, coupling constant τ_T_ = 1 ps) and a Parrinello–Rahman barostat (*p* = 1 bar, τ_p_ = 12 ps, compressibility β = 3 × 10^4^ bar^–1^),^52^ respectively. Table S2 shows a summary of the unbiased CG MD simulations performed in this work. Simulation times of individual systems ranged between 25 and 40 μs, for a total of 620 μs, where each system was replicated twice (Table S2).

#### AA Simulations

Subsequently, for a detailed analysis of the ligand-binding poses in SBD1, we backmapped selected conformations obtained from the CG MD simulations with the ligand bound in the binding site and used these as a starting point of subsequent all-atom (AA) MD simulations. The structure backmapping was performed using the *backward.py* script.^53^ The backmapped systems were simulated using the CHARMM36 force field^54,55^ with the TIP3P^56^ water model. The backmapped protein was placed in a cubic box of 10.0 × 10.0 × 10.0 nm, solvated by ∼35,363 waters, and neutralized, and 0.15 M NaCl was added. After solvation, each system was subjected to energy minimization using the steepest descent algorithm until the maximum force of 1000 kJ mol^–1^ nm^–1^ was achieved. The systems were optimized and equilibrated for at least 1 ns in the NVT ensemble and 10 ns in the NPT ensemble. After the systems were simulated for 100 ns twice in the NPT ensemble with the Nose–Hoover thermostat^57^ and the Parrinello–Rahman barostat^52^ with a reference temperature and pressure of 303.15 K and 1 bar, respectively. The nonbonded interactions were treated using the Verlet cutoff scheme, with the particle mesh Ewald (PME) method^58^ to treat long-range electrostatic interactions, while the short-range electrostatic and van der Waals interactions were calculated with a (real space) cutoff of 12 Å. Periodic boundary conditions were applied to all simulations, and bonds involving hydrogen atoms were constrained by using the linear-constraint-solving (LINCS) algorithm.

#### MD Analysis

**Figure 2:**
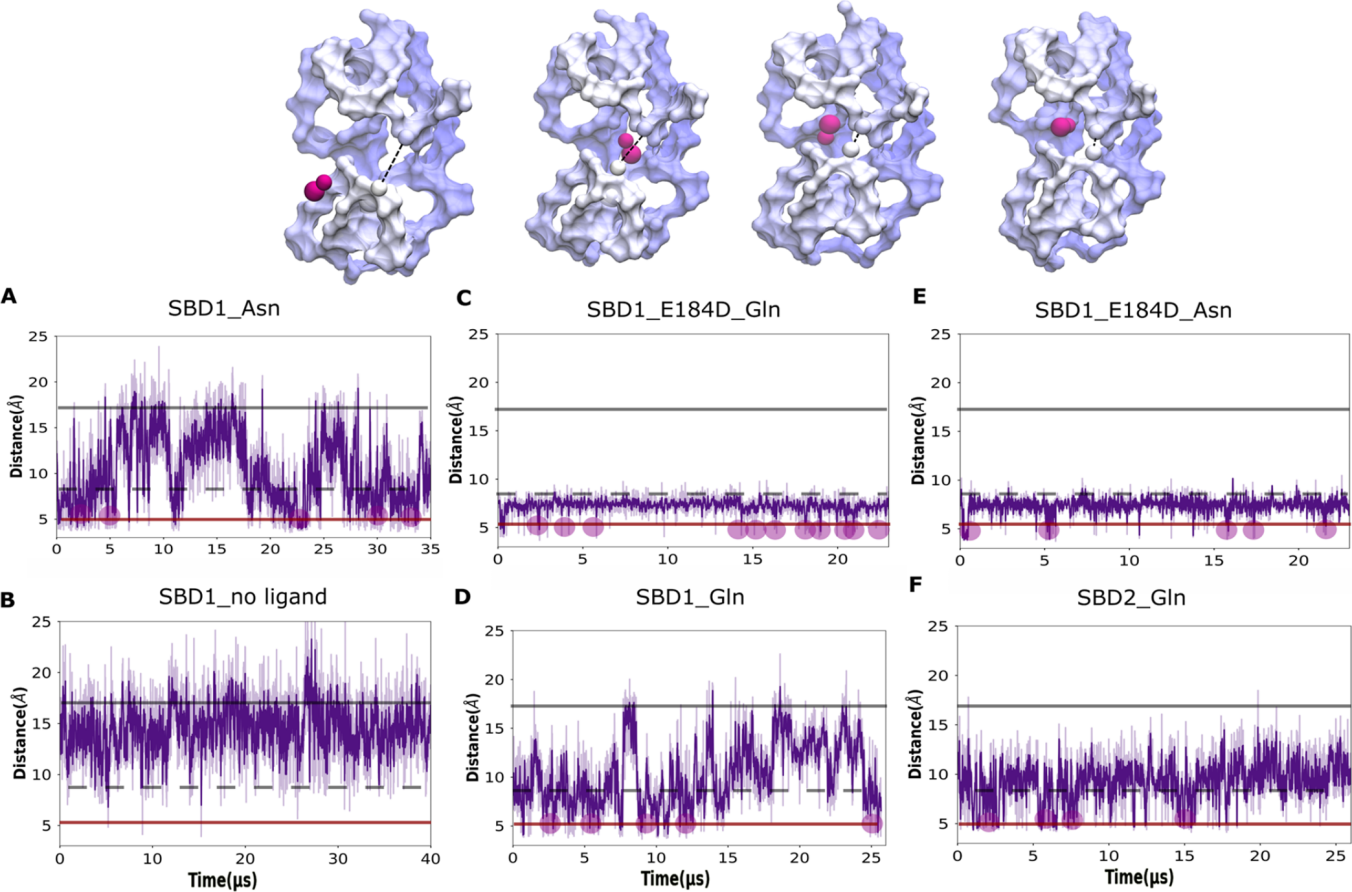
Opening-closing transitions of SBD observed with CG MD. The temporal evolution of the monomer distances based on the center of geometry of the D1 and D1 subdomain residues of SBDs as described in the Methods section were analyzed. SBD1 wild-type in the presence of Asn (A) with snapshots representing the closure of the SBD1 domain at the moment of ligand binding (Asn in vdW representation, magenta), in the absence of a ligand (B) in the presence of Gln (D), SBD1 E184D mutant in the presence of Gln (C) and Asn (E), and SBD2 wild-type with Gln (F). Straight lines limit distances between subdomains at ∼5 Å (closed state as obtained from our CG MD simulations, in red) and ∼17 Å (open state, as observed in the crystal structure in the *apo* form (PDB ID: 4LA9), in gray), respectively. The gray dashed line represents a semiopen state. The magenta circles represent the protein’s closure upon binding to the ligand. Probability distributions of obtained distances are shown in the Supporting Information, Figures S4 and S5.

We analyzed the temporal evolution of the monomer distances based on the center of geometry of the D1 subdomain residues (10–11) to the center of geometry of the D2 subdomain residues (137–138) for SDB1 and SBD1 E184D mutants (Figure 2). In SBD2, distance profiles were calculated between the center of geometry of the D1 subdomain residues (61–62) and the center of geometry of the D2 subdomain residues (128–129). Visual inspection of the trajectories was performed with VMD^59^ and PyMOL (DeLano Scientific, Palo Alto, CA).

## Results

### Crystal Structure-Overall Organization and Ligand Binding

We crystallized the SBD1 of ABC importer GlnPQ from *L. lactis* in the presence of asparagine and an excess of arginine and solved these structures at 1.7 and 1.6 Å resolution, respectively (PDB IDs: 6FXG, 8B5E). In addition, we obtained glutamine-bound crystals of the SBD1 (E184D) mutant that yielded a structure at 2.0 Å resolution (PDB ID: 8B5D) (see Table 1 for data and refinement statistics). Due to the relatively high-resolution and the fact that all of the structures revealed a similar architecture of the binding sites, the reliable analysis of possible structural differences, which potentially could explain changes in the ligand affinity, seems feasible. The bound substrates form a vast network of interactions. The α-carboxyl group of the bound Asn interacts with R101 and with the backbone nitrogen atoms of T96, A144, and A145 (via water) from the large and small subdomains, respectively (Figure 1C). The α-amino group makes hydrogen bonds via water to the Oδ2 atom of E184, the hydroxyl side chain of Y38, and the backbone carbonyl of G94. The side chain moiety of bound asparagine is sandwiched in a hydrophobic pocket formed between Y38 and F76. The Nε2 atom of the asparagine forms hydrogen bonds with the Oδ2 atom of D35 and the backbone carbonyl of A93, whereas the Oε1 atom of the asparagine makes direct hydrogen bonds to the Nζ of K140. In addition, the Oε1 atom makes hydrogen bonds via one water molecule with the Oδ1 of D183 and via two water molecules with the backbone nitrogen of the A145 side chain, respectively.

An interesting structural difference is observed when the wild-type (WT) SBD1 is compared with the E184D mutant, which has ∼90-fold increase in the affinity for Gln and an almost 10-fold decrease in affinity for Asn^24^ (see Table S1). The shorter side chain of D184 induces a different orientation of the hydroxyl groups (Figures 1D and S2A), which mimics D417 in SBD2. Furthermore, the α-amino of the Gln ligand is differently positioned and shows hydrogen bonding to the hydroxyl group of T96 (like 28 in SBD2) and the Oδ2 of D184 (like D417 in SBD2). The hydrogen bonding with the backbone carbonyl of G94 is the same in the SBD1(E184D) mutant and wild-type SBD2 (G326 in SBD2). In asparagine-liganded SBD1, Y38 makes a hydrogen bond to the α-amino group of asparagine, which is different in SBD1(E184D) where the interaction of the α-amino group is made by the Oδ2 of D184 and D183.

The effect of nontransported substrates on substrate affinity was shown using FRET measurements, where arginine, histidine, and lysine can competitively inhibit the uptake of glutamine (via SBD1 and SBD2) and asparagine (via SBD1).^35^ To investigate this inhibitory effect, we aimed to obtain the crystal structure of arginine bound to SBD1. Unfortunately, we failed to obtain well-diffracting SBD1-Arg crystals; hence, we soaked crystals of the unliganded SBD1 with 10 mM arginine (Figure 1E and Table 1). In the obtained crystal structure, there are two molecules of SBD1 in the asymmetric unit cell, which show a slightly different binding pattern of arginine. Most interactions are with the large subdomain and the carboxyl-side chain of E184, which makes a hydrogen bond to the α-amino group of the Arg. Similar to the binding of the high-affinity substrate asparagine, the α-carboxyl group of arginine is stabilized by a salt bridge with R101 and hydrogen bonds to the backbone nitrogen and the hydroxyl group of T96. Clearly, the bound arginine traps SBD1 in a conformation that is unable to close, hence explaining the inability of GlnPQ to transport arginine leading to the inhibition of the transport of Gln and Asn. It is also notable that this binding does not affect the global rearrangement of the protein structure (Figure S6). One might argue that possible conformational changes (from open to close conformation) cannot be tolerated by crystal lattice, but such conformational changes *in crystallo* have been observed for another glutamine-binding protein, namely GlnBP.^60^ Furthermore, partial domain closure has also been demonstrated for ligand-binding domains of ionotropic glutamate receptors iGluRs.^61^ Together with the fact that also in solution arginine prevents closure of SBD1,^35^ we believe that our observation that arginine is incapable of triggering the conformational change is correct.

### Molecular Dynamics Simulations

To further characterize the differences in the affinity of the ligands of SBD1, we performed coarse-grained molecular dynamics (CG MD) simulations using the Martini 3 force field^42,62^ in combination with the Go̅-like model^44^ on the newly resolved crystallographic structure of SBD1 (PDB ID: 8B5E). The use of the CG Martini model in combination with Go̅-like interactions allows to sample long time scale protein dynamics, including spontaneous binding of ligands, as demonstrated previously.^63−66^

To simulate spontaneous ligand binding, Arg was removed from the binding site, whereas other ligands (either Asn, Gln, Arg, or His) were randomly placed in the surrounding solvent, with one ligand per system. To improve the sampling of many binding/unbinding events, the ligands were restricted to remain in the vicinity of the binding site (see Methods for the details). We also performed the CG MD simulation on SBD1 E184D using the obtained crystallographic structure (PDB ID: 8B5D) and the SBD2 domain in the ligand-free state (PDB: 6H30) to study the changes in the ligand-binding affinity caused by the introduced mutation in SBD1. Table S2 summarizes all of the CG MD systems simulated in this work.

#### Implication of Global and Local Motions

In all instances of the CG MD simulations on SBD1 with the Arg ligand removed from the binding pockets between the D1 and D2 subdomains, we observed SBD1 opening and closure events, confirming the protein flexibility necessary for the recruitment and binding of new ligands from the bulk. This is evident from the temporal analysis of the binding distance between D1 and D2 (Figure 2). We also observed spontaneous transitions to the closed state from the open state independent of the presence of a ligand in the simulation box (Figure 2, SI Movie S1), which correlates with an earlier MD study of the SBD2 domain.^34^ Global subdomain shift to an open state already begins during the first hundreds of nanoseconds of simulation; during the simulations, the closed domain arrangement of SBD1 eventually destabilizes and adopts a global open conformation. When compared with the crystal structure in the apo form (PDB ID: 4LA9), with the interdomain distance ∼17 Å, a wider opening is observed in simulations with the distance between subdomains reaching ∼22 Å (Figure 2). As anticipated, the most significant structural rearrangements in SBD1 take place in the flexible hinge region, where the main bending and unbending occur due to the rotational movement of the domains, as shown schematically in Figure 1B. The details of open-close transition rearrangements are presented in the Supporting Information, Figures S7 and S8.

To investigate how the opening/closing transition observed for the WT SBDs domains is correlated to the binding of the ligands when these are present, we analyzed the time traces of the distance between the ligands and the binding pocket. Figures S2 and S3 summarize the obtained results for the four examined ligands l-Asn, l-Gln, l-Arg, and l-His. We find that all ligands start diffusing from the bulk and eventually arrive at the binding site followed by a subdomain closure, known as the Venus-fly trap mechanism.^24,35^Movie S4 demonstrates a representative simulation trajectory of SBD1-Asn recognition and the binding process in the coarse-grained setup. The interaction of the ligand with the protein occurs on both sides of the binding pocket. The entering ligand alternately forms interactions with the polar residues of the pocket, such as Asp8, Ser10, Thr116, Asp138, Asp156, and Glu157, and forms interactions between subdomains until the subsequent complete closure of the binding pocket occurs.

The final simulated bound state in the SBD1-Asn system, as obtained from the current coarse-grained model after backmapping (Figure 1B, top), is in good agreement with the determined Asn-bound crystallographic structure (PDB ID: 6FXG) with the root-mean-square deviation (RMSD) of ∼0.6 Å (Figure S2C). Figure S3B shows the Asn probability density around SBD1, where the higher density is observed inside the binding pocket, and only a small area of density is observed between domains (blue isosurface).

In the case of Gln, a high density is also observed in the main binding site but an increase in the size of the binding cavity and spontaneous binding can be observed, which may indicate less stable ligand binding (Figure S2). In addition, compared to asparagine, there is an increase in the distance between subdomains (Figure 2B), and no complete closure of the domain in the presence of the ligand at the binding site can take place. Partial domain closure in the presence of glutamine was also demonstrated previously.^35^ Moreover, the frequency of a domain transition from an open state to a closed state increases (Figure 2A). The differences in opening and closing rates in the presence of the ligands are in line with earlier reports.^24^ Besides, Asn has more interactions with the protein, which can affect the binding strength. Figure S3A shows the structures of SBD1 wild type in the presence of Asn and Gln ligands, obtained after CG MD simulation followed by Martini backmapping and performing all-atom MD simulation (see Methods for details). This provided a comprehensive depiction of domain binding at the binding sites. On the time scales of conformational changes, rearrangements in the hydrogen bonding network are relatively fast. Thus, all of this may have an effect on the strength and rate of binding, which may also explain the lower affinity binding of glutamate to the SBD1 domain.

Using a competition ITC, it was shown that the affinity of the substrates follows: Asn > Gln > His > Arg preference according to the *K*_D_ values. In addition, arginine binds in the substrate pocket but is not transported by GlnPQ.^35^

To characterize the binding of low-affinity substrates (*K*_D_ > 10 μM), we performed CG MD simulations in the presence of Arg and His. The binding densities of the ligands around SBD1 are depicted in the Supporting Information, Figure S3. The binding pocket in the presence of Arg shows the highest occupancy, in agreement with the crystal structures of SBD1 soaked with Arg (PDB ID: 8B5E). Interestingly, in the presence of His, most of the binding density is in the interior part of the binding pocket, which may also interfere with tight closure of the subdomains (Figure S3B,C). More details about the closing states can be obtained by analyzing the distances between the opposite subdomains D1 and D2 over the MD trajectories, as shown in the Supporting Information, Figure S3B. For both ligand-bound systems (SBD1-Arg and SBD1-His) (Table S2), the measured distance between subdomains was 6–8 Å, which is larger in comparison with the situation when the preferred ligands Asn and Gln are bound (4.5–5 Å), showing that the full closure of the binding pocket is not observed, leading to a reduced affinity. The Supporting Information, Figure S3A,B, shows the different snapshots of Arg and His conformations in the binding pocket obtained during our CG MD simulations. This observation is supported by the loss of transport activity due to its inability to fully close and bind high-affinity substrates.

To check whether there are any differences in the behavior of the two SBDs, we performed an additional CG MD simulation of SBD2 in the open state taken from the tandem SBDs X-ray structure (PDB ID: 6H30). The protein also showed high global and local motions of subdomains in our MD simulations in Figures 2 and S1. Switching between the two conformations in SBD2 also involves a hinge region, but unlike SBD1, it is a reorientation of the subdomains relative to each other at a 50–60° angle (Figure 2, SI Movie S3).

Next, to visualize the conformational changes upon ligand binding in each of the domains of the tandem, we performed CG MD simulations of the SBD1–SBD2 tandem of GlnPQ (PDB ID: 6H30) in the presence of asparagine and arginine (SBD1 SBD2-Asn and SBD1 SBD2-Arg systems, Table S2). The tandem arrangement of the domains has no significant impact on domain mobility during the transition from an open to closed state, including the ligand-binding process. However, certain preferred arrangements of domains were observed.

For the closed state of the SBD2 domain, we noted two considerable conformations: (i) SBD1 is adjacent to the upper part of SBD2, where the linker is shortened due to folding and subsequent juxtaposition of domains due to the formation of the cross-domain interactions (Asn442-Ser179, Lys227-Gly271); (ii) the separation of domains due to the linker length rearrangements with the linker-SBD2 interactions (Lys227-Gly211), where domain 1 moves into the perpendicular position relative to domain 2 (Figure S1, left). Our data support the suggestion that SBDs may be stabilized by direct protein–protein interactions proposed earlier.^26^ There is also a peculiar arrangement of domains in an open state for both SBDs, resembling a butterfly, where SBD1 comes close to SBD2 (Tyr163-Tyr220 interaction and convergence of domains in Asp96-Lys307) and subdomain 1 of the SBD1 domain approaches the hinge region of SBD2 (Figure S1, right).

#### Impact of E184D Mutations on the Dynamics of SBD1

To investigate the profound effect of the E184D mutation on the affinity for Gln, we performed CG MD simulations and studied the binding of Asn and Gln to the E184D single mutant of SBD1 and wild-type SBD2. Interestingly, in the E184D mutant, such a significant domain transition from the closed to open state was not observed and the subdomain distance reached only 12 Å maximum, which can be referred to as a semiopen state (Figures 2, 1B and Movie S2). It is also interesting to note the increased frequency of domain closure, which is in agreement with the experimentally reported increased mobility of SBD1 E184D mutant in the presence of Gln.^24^

In line with the experimental results, an increase in the surface of the binding pocket and a corresponding increase in the number of contacts with water inside the pocket are observed in the obtained all-atom structure (Figure 1C,D), which is also observed for the SBD2. As in the wild type, initial ligand-protein interactions occur with polar residues (Ser, Thr, and Asn) on both subdomains, which subsequently lead to complete domain closure (Movie S1). The difference is observed in the increased frequency of opening and closing of subdomains during ligand binding due to a decrease in the distance between subdomains in the open state of the E184D mutant (Figure 2A). Figure S2B shows the probability densities for the Gln and Asn ligands at the binding pocket (transparent blue isosurface). The density representing Gln binding in E184D_SBD1 is very similar to that of Asn in SBD1 (Figure S2B), whereas for Asn, a decrease in the binding surface is observed. The observed binding position of the ligand in the binding pocket is the same as that observed in the crystallographic structure (Figure S2A). After a comparison of binding sites obtained from CG MD simulations, we performed all-atom MD simulations on the E184D SBD1 mutant as well as on WT SBD2 (Figure S2A). We analyzed the number of contacts between the active site of SBD1 and water molecules, and the E184D mutant shows more such contacts when compared to the WT SBD1-Gln (bottom panels in Figure 1D,E).

## Discussion and Conclusions

Despite the numerous studies of the conformational dynamics in ABC transporters, some intricate details are still elusive. The ABC transporter GlnPQ from *L. lactis* has two covalently linked substrate-binding domains (SBDs), and due to its inherent flexibility, we have chosen it as a suitable model to study the dynamics of the SBDs.

By combining X-ray crystallography and CG MD simulations, we explored the conformational dynamics of the SBD1 domain of GlnPQ and its ligand specificity. We demonstrate that the SBDs can convert from open to closed ligand-free conformation. The MD simulations suggest that the ligand binding to the SBD1 domain is a two-step process, where, in the first step, the initial interaction between the protein and the ligand is established; subsequently, in the second step, after several ligand transitions within the binding pocket between subdomains, the conformational changes of the subdomains lead to the closure of the ligand-binding site. Interestingly, the tandem domain arrangement does not affect ligand binding, as both single domains and tandem show similar ligand interactions, eventually leading to similar closed conformations in the presence of high- and low-affinity ligands. This is in line with the ITC and in solution smFRET experiments.^26^

Furthermore, the mobility of the linker in the GlnPQ transporter provides additional flexibility to both SBDs; we observed different orientations of them and noted two main arrangements (Figure S1). It is important to note that the actual length of the linker is also important for the efficient delivery of substrates from SBDs to the translocon. This is based on the observation of numerous linker-SBD interactions during domain movements relative to each other. Overall, considering the position of the linker between the SBDs, the observed interactions during the binding process, and the importance of the linker length (deleting eight amino acids diminishes asparagine uptake by more than 90%),^68^ we hypothesize that the linker mobility has a stronger effect on the substrate delivery by SBD1 than by SBD2.

Moreover, we observed different conformational dynamics of SBD1 in the presence of various ligands. The main difference was found in the domain closure upon ligand binding. In the presence of l-glutamine, a partial closure and more pronounced initial binding are observed compared to l-asparagine. The presence of l-arginine and l-histidine at the binding site leads to the inability of SBDs to close fully hence leading to the loss of transport activity of the transporter.

The herein reported structure of the SBD1 E184D in complex with l-Gln, combined with the MD simulations, explains the change in affinity for Gln when compared to the wild-type protein. While the global architecture of the binding pocket remains unchanged, there are differences in protein–ligand interactions. The shorter side chain of aspartate at position 184 induces a different orientation of the hydroxyl groups creating additional space for glutamine to bind with a high affinity, but almost without any impact on the affinity for asparagine binding. According to the obtained experimental and MD simulations results, it can be assumed that an increase in the size of the binding pocket, and a decrease in the distance between subdomains in the open state (semi- open state), accelerates the stabilization of the binding site and reduces the transition time to the final closed state; i.e., the SBD1 domain becomes more specific in the ligand binding, which is reflected in the *K*_D_ value (Table S1).

In summary, the obtained crystal structures and performed MD simulations fill the gaps in the understanding of mechanistic details of ligand recognition and SBD dynamics. This study can serve as an example for the further characterization of other ABC transporters with mobile SBDs.

## Data Availability

The coordinates of the refined models and structure factors have been deposited into the PDB repository: 6FXG for SBD1-Asn, 8B5D for SBD1(E184D)-Gln, and 8B5E for SBD1-Arg. Models and parameter files used for MD simulations are freely available from the Zenodo Web site at the following url: 10.5281/zenodo.10049053

## Supporting Information Available

Binding characteristics of SBDs determined by ITC; snapshots of the conformations of the SBDs domains of the tandem; binding sites of SBDs after CG MD simulations; analysis of the AA MD simulations; distance profiles of SBDs in the presence of Arg and His; probability distributions of obtained distances between subdomains in CG MD simulations (PDF)
Transitions of WT SBD1 protein 1 from the open to the closed state (Movie S1) (MPG)
Transitions of the E184D mutant from the open to the closed state (Movie S2) (MPG)
Transitions of WT SBD2 protein 1 from the open to the closed state (Movie S3) (MPG)
WT SBD1-Asn recognition and the binding process (Movie S4) (MPG)

## Supplementary Material

jp4c02662_si_001.pdf

jp4c02662_si_002.mpg

jp4c02662_si_003.mpg

jp4c02662_si_004.mpg

jp4c02662_si_005.mpg
