# Supplementary material for: Exploring the Ligand Binding and Conformational Dynamics of the Substrate-Binding Domain 1 of the ABC Transporter GlnPQ
Source: J Phys Chem B. 2024 Aug 2;128(32):7822–32. doi: 10.1021/acs.jpcb.4c02662 (PMC11331510; doi:10.1021/acs.jpcb.4c02662)
Supplement: Supplementary file 1 — jp4c02662_si_001.pdf [file jp4c02662_si_001.pdf]

Supporting Information for Exploring the Ligand Binding and Conformational Dynamics of the Substrate Binding Domain 1 of the ABC Transporter GlnPQ

M. Nemchinova[1#], G.K. Schuurman-Wolters[1#], J.J. Whittaker[1], V. Arkhipova[1], S. J. Marrink[1], B. Poolman[1], A. Guskov[1]*

[#]Equal contribution
[1]Groningen Institute for Biomolecular Sciences and Biotechnology, University of Groningen, 9747AG Groningen,
The Netherlands
*Correspondence to a.guskov@rug.nl

Contents

SUPPLEMENTARY NOTE 1: **1**
- Table S1.Binding characteristics of SBDs determined by ITC. 1
- Table S2. Summary of all unbiased CG MD simulations of GlnPQ protein performed in this work. 2
- Figure S1. 3
- Figure S2. 6
- Figure S3. 7
- Figure S4. 7
- Figure S5. 7
- Figure S6. 8
- Figure S7. 8
- Figure S8. 9
- Supplementary SI Movie 1. 9
- Supplementary SI Movie 2. 9
- Supplementary SI Movie 3. 9
- Supplementary SI Movie 4. 10

SUPPLEMENTARY REFERENCES **10**

SUPPLEMENTARY NOTE 1:

Table S1. Binding characteristics of SBDs determined by ITC.

Errors are the standard deviation between 2-5 independent experiments.

Domain name	ΔG (kJ.mol^{-1})	n	KD (μM)	ΔH (kJ.mol^{-1})	TΔS (kJ.mol^{-1})
Asn (SBD1)					
SBD1#	0.69±0.1	0.2 ± 0	-73.6 ± 1.2	-34.9 ± 1.1	-38.2 ±1.6
SBD1-2#	0.84 ± 0.03	0.4 ± 0.08	-74.8 ± 2.8	-38 ± 3.3	-36.8 ± 0.7
SBD1(E184D)	0.53 ± 0.02	1.7 ± 0.14	-58.7 ± 1	-25.8	- 32.9
SBD1(E184D_V185D)*	0.54 ±0.02	2.4 ± 0.35	-57.3 ± 3.4	-25.4	-31.9
SBD1-2 (D417F)	0.86 ± 0.1	0.4 ± 0.06	-73 ±0.4	-37 ± 4	-37 ± 0.4
SBD1 (E184W)	No affinity				
SBD1 (Y38F)	0.74 ± 0.08	0.2 ± 0.01	-77.3 ± 7.3	-39	-38.3
Gln (SBD1)					
SBD1	0.98 ± 0.12	92 ± 16	-23.9 ±6.3	-0.9 ± 0.1	-23.0 ± 7.1
SBD1-2**	0.79 ± 0.03	181 ± 98	-57.7 ± 15.6	-36 ± 75	-21.7 ± 1.7
SBD1(E184D)	0.53 ± 0.02	1.0 ± 0.1	-65.3 ± 0.6	-30.9	-34.4
SBD1(E184D_V185D)*	0.58 ±0.02	1.3 ± 0.15	-65.7 ± 2.6	-31.9	-33.8
SBD1-2 (D417F)	0.8 ± 0.07	194 ± 37	-33 ± 3	-12 ± 4	-21 ± 0.5
SBD1 (E184W)	No affinity				
SBD1 (Y38F)a	0.78 ± 0.01	127 ± 20	-35.5		

Gln (SBD2)					
SBD2	0.75 ± 0.07	0.9 ± 0.2	-19.3 ± 2.2	15.4 ± 0.7	-34.7 ± 2.4
SBD2(D417F)	No affinity				
SBD1-2b	0.78 ± 0.04	0.6 ± 0.2	-20.1 ± 0.3	15.4 ± 1.3	-35.5 ± 1.2
SBD1-2(E184W)	0.78 ± 0.01	1.5 ± 0.8	-22.2 ± 2.7	11 ± 4.2	- 34.2 ± 2
Gln (SBD1-2)					
SBD1-2(E184D_V185D)*	1.33 ± 0.02	0.65 ± 0.2	-43.2 ± 0.3	-7.8	-35.4

*a Experiment performed in the presence of 1 mM Gln titrated with 250 µM Asn (so-called competition ITC); [b] determined using conditions where SBD1 is not titrated; *The experiment has been done once, therefore no error on TΔS and ΔG; **For calculating the SBD1 binding values the parameters for SBD2 were fixed in the origin fitting program. # Data have been taken from previous publications [1–5].*

Table S2. Summary of all unbiased CG MD simulations of GlnPQ protein performed in this work.

System name	PDB ID	System's simulation length (µs)	Box size, nm	Total beads
SBD1_no_ligand	8B5E	40	15x15x15	30178
SBD1-Asn	8B5E	35	15x15x15	30178
SBD1-Gln	8B5E	25	15x15x15	30178
SBD1-Arg	8B5E	28	15x15x15	30178
SBD1-His	8B5E	32	15x15x15	30178
SBD1_E184D-Asn	8B5D	25	15x15x15	30153

SBD1_E184D-Gln	8B5D	25	15x15x15	30153
SBD1_E184D	8B5D	3	15x15x15	30153
SBD2-Gln	6H30	25	15x15x15	29935
SBD1_SBD2-Asn	6H30	40	20x20x20	69259
SBD1_SBD2-Arg	6H30	40	20x20x20	69261

Cumulative time: 620 μs.

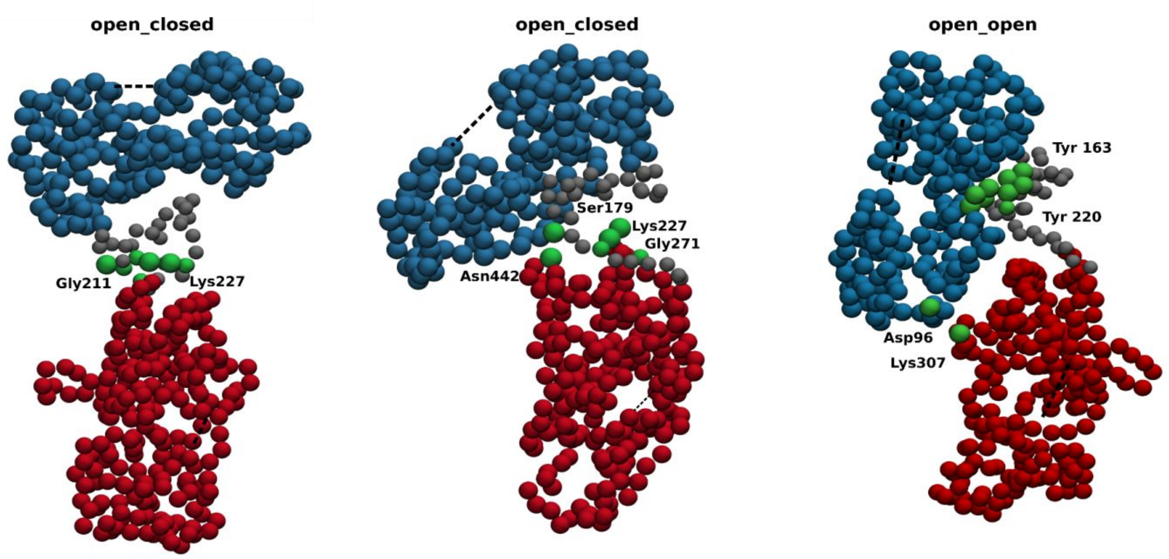

Figure S1. Snapshots of the conformations of the SBDs domains of the tandem relative to each other in the closure of one of the domains (left, middle) and the opening of both domains (right) obtained after CG MD simulations.

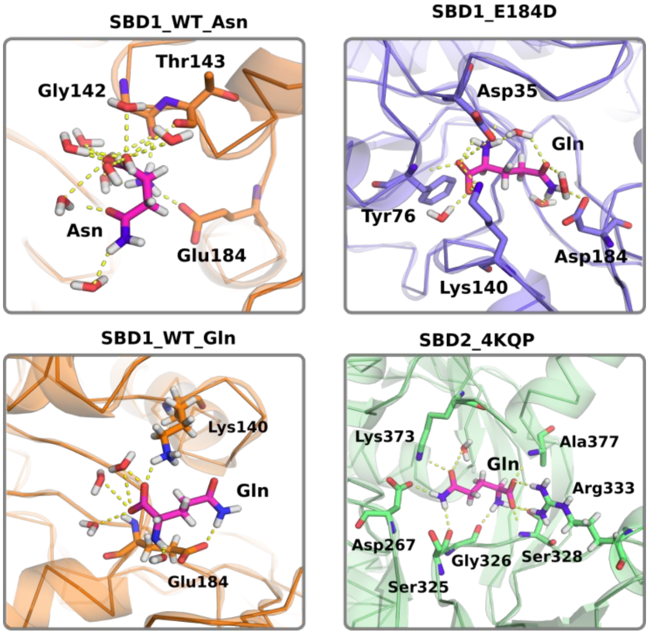
SBD1_WT_Asn
Thr143
Gly142
Asn
Glu184
SBD1_E184D
Asp35
Gln
Tyr76
Asp184
Lys140
SBD1_WT_Gln
Lys140
Gln
Glu184
SBD2_4KQP
Lys373
Ala377
Gln
Arg333
Asp267
Gly326
Ser328
Ser325
A

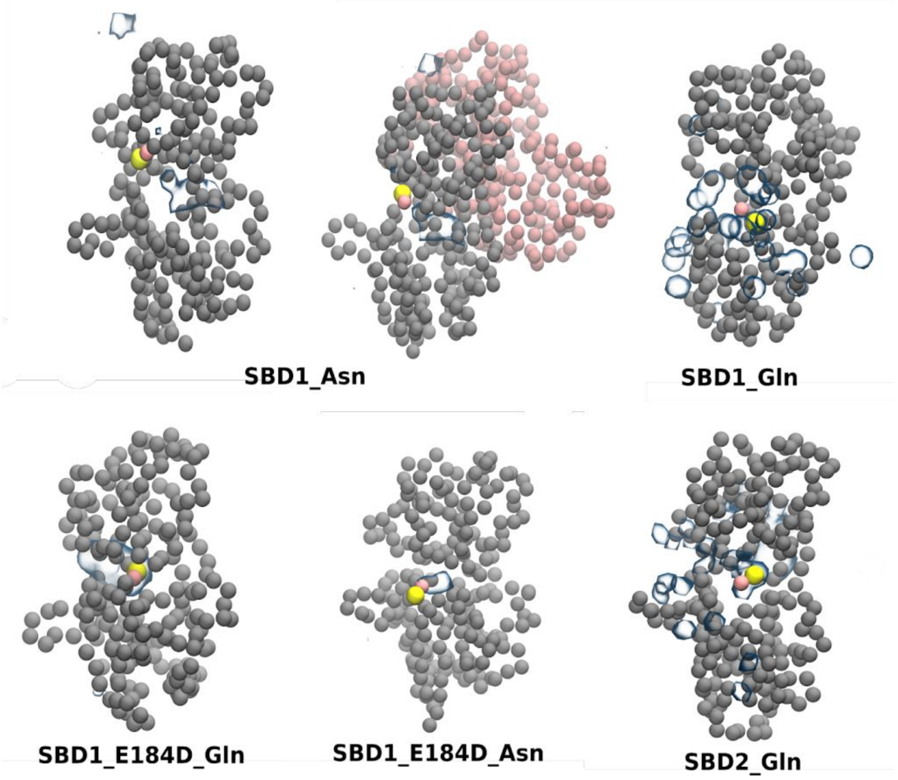
SBD1_Asn
SBD1_Gln
SBD1_E184D_Gln
SBD1_E184D_Asn
SBD2_Gln
B

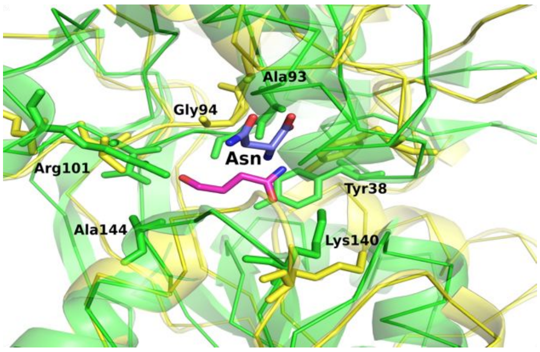
Ala93
Gly94
Asn
Arg101
Tyr38
Ala144
Lys140
C

Figure S2. (A) Binding sites of SBDs after CG MD simulations. Binding residues labelled with numbers have interactions with the substrate (in stick representation). The ligands are shown in magenta. Oriented water molecules are shown in stick representation and these have interactions with residues important for ligand binding. The dashed lines show polar contacts in the binding sites, which range from 1.8 Å to 3.6 Å. **(B)** Ligand densities around SBD1, SBD1_E184D mutant and SBD2 of the GlnPQ transporter obtained from averaging unbiased CG MD simulations. The blue isosurfaces correspond to a 1,000 fold higher ligand density than in solution. **(C)** Overlay of the crystallographic structure (PDB ID: 6FXG, in green) with the one simulated bound state of SBD1-Asn (in yellow). Aligning the structures gives a RMSD of ~0.6 Å. Binding residues labelled with numbers have interactions with the substrate (in stick representation). Residues interacting with Asn are labeled by number.

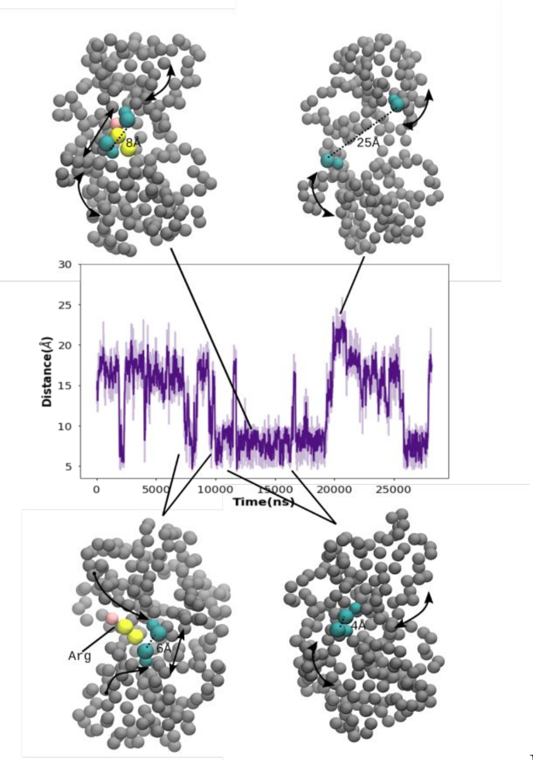

A

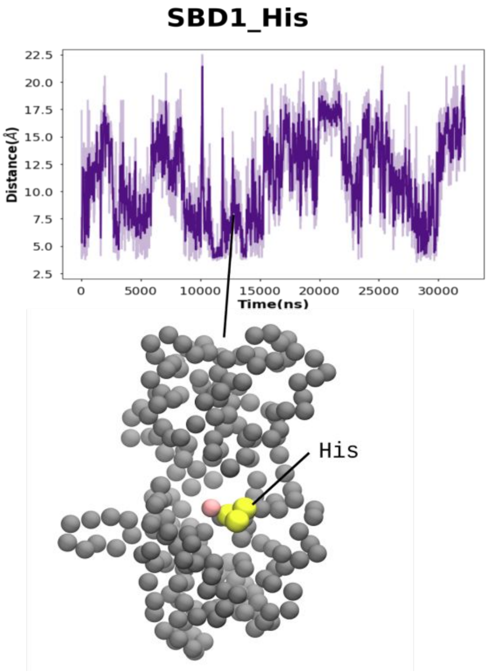

B

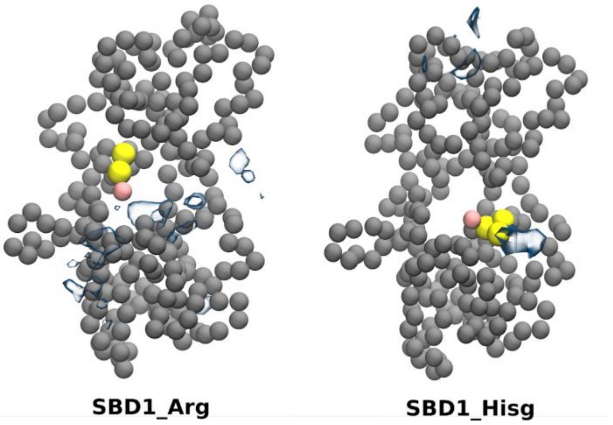

C

Figure S3. Distance profiles with snapshots of SBDs between the center of geometry of the D1 subdomain (10-11 residues) to the center of geometry of the D2 residues (137-138 residues) in the presence of the low affinity substrates - Arg **(A)** and His **(B)**. **(C)** Ligand densities of Arg and His around WT SBD1 obtained from averaging unbiased CG MD simulations. The blue isosurfaces correspond to a 1,000 fold higher ligand density than in solution.

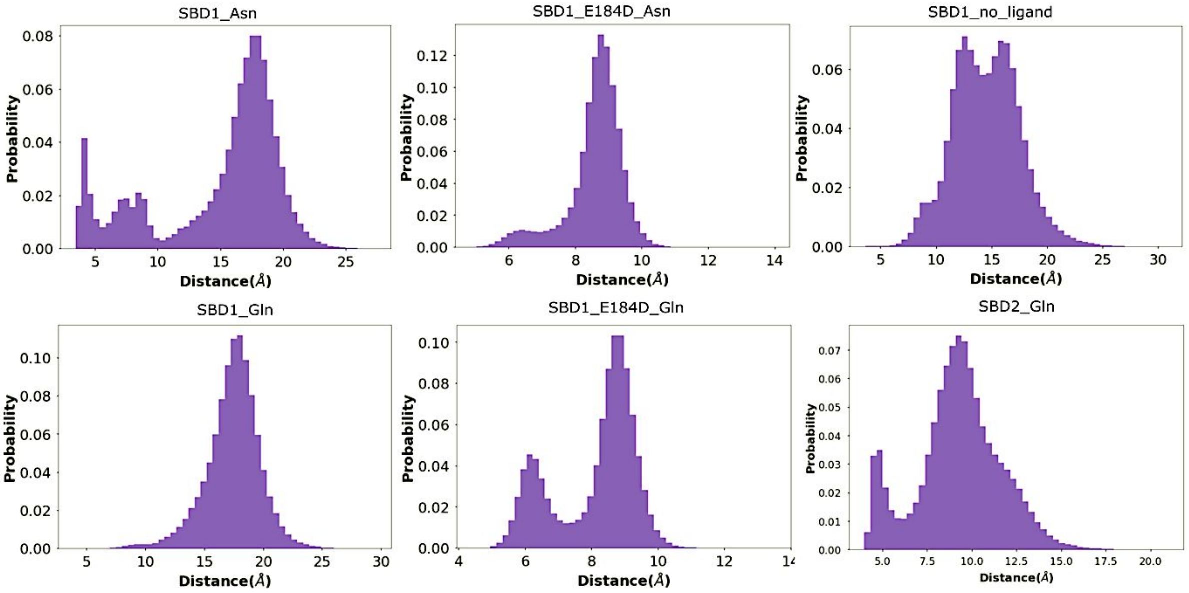

Figure S4. Probability distributions of obtained distances in Fig.2 in the main text between subdomains in CG MD simulations of SBD1 wild type, SBD1 E184D mutant and SBD2 in the presence of Gln and / or Asn ligands.

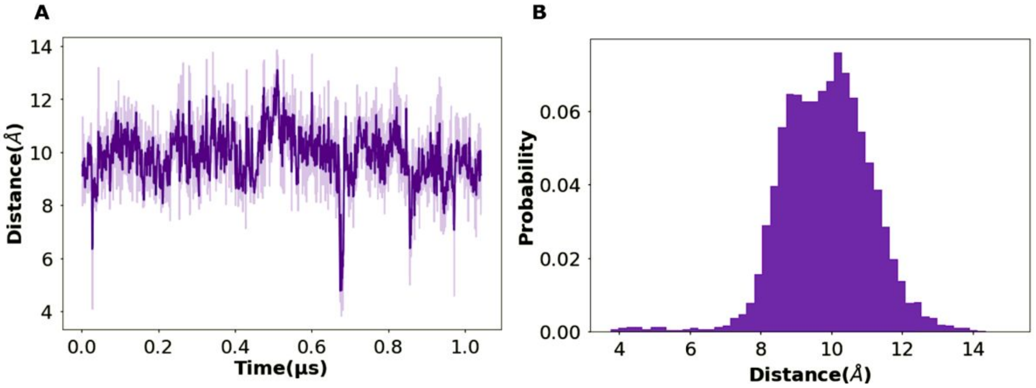

Figure S5. Opening-closing transitions of SBD1_E184D mutant observed with CG MD simulations. Distance profiles were calculated between the centers of geometry of the D1 and D2 subdomains in the absence of the ligands and probability distributions of obtained distance. For the CG MD simulation in the presence of the ligand, the protein model was used after 1 μs of CG MD simulation in the absence of the ligand, which was carried out after 500 μs of preliminary MD CG simulation.

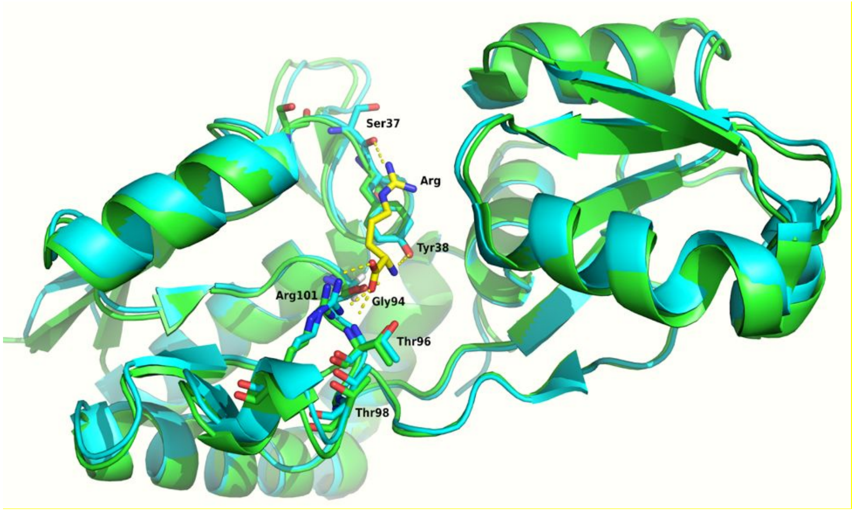

Figure S6. Superposition of unliganded and Arg-bound structures of SBD1. Two structures (PDB ID 4LA9 (green) and 8B5E (cyan), respectively) were used. Overlay of the residues in the active site was done by aligning the hinge region as an anchor point. Aligning the structures gives a RMSD of ~0.23 Å. Residues interacting with arginine are labeled by number. The dashed lines show polar contacts in the binding sites, which range from 1.8 to 3.6 Å.

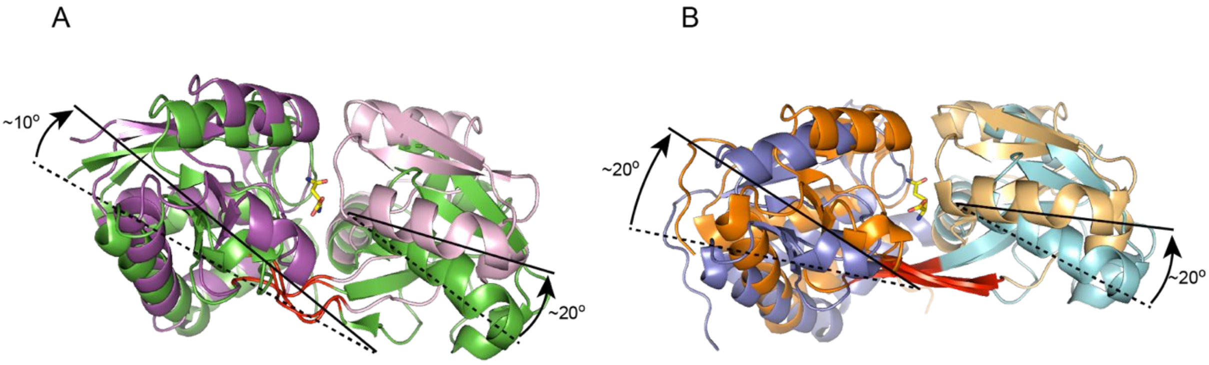

Figure S7. Superposition of liganded and unliganded structures to show the venus-fly trap movement of the SBDs upon binding of a substrate. Sequences were aligned using the hinge region as anchor point (shown in red). (A) shows the subdomain motions upon binding of asparagine to unliganded SBD1 (green, open unliganded; purple, closed-liganded). (B) shows the subdomain motions of SBD2 upon binding of glutamine (blue, open-unliganded; orange, closed-liganded. Binding of substrate results in movement of the domains relative to each other as shown in Figure 1B. For SBD2 this movement displaces the lobes relative to the other more than for SBD1, where this relative movement is smaller and best described by a twisting/rotational movement. This difference might relate to a different structure of the linker between the lobes; for SBD2 this linker is a clear double βeta-sheet, for SBD1 the linker has more flexibility. Polar interactions between SBD1 D35 (SBD2 D267) from the large lobe, the substrate and SBD1 K140 (SBD2 K373) of the small lobe probably play a role in the closure of the SBDs (Fig. S8).

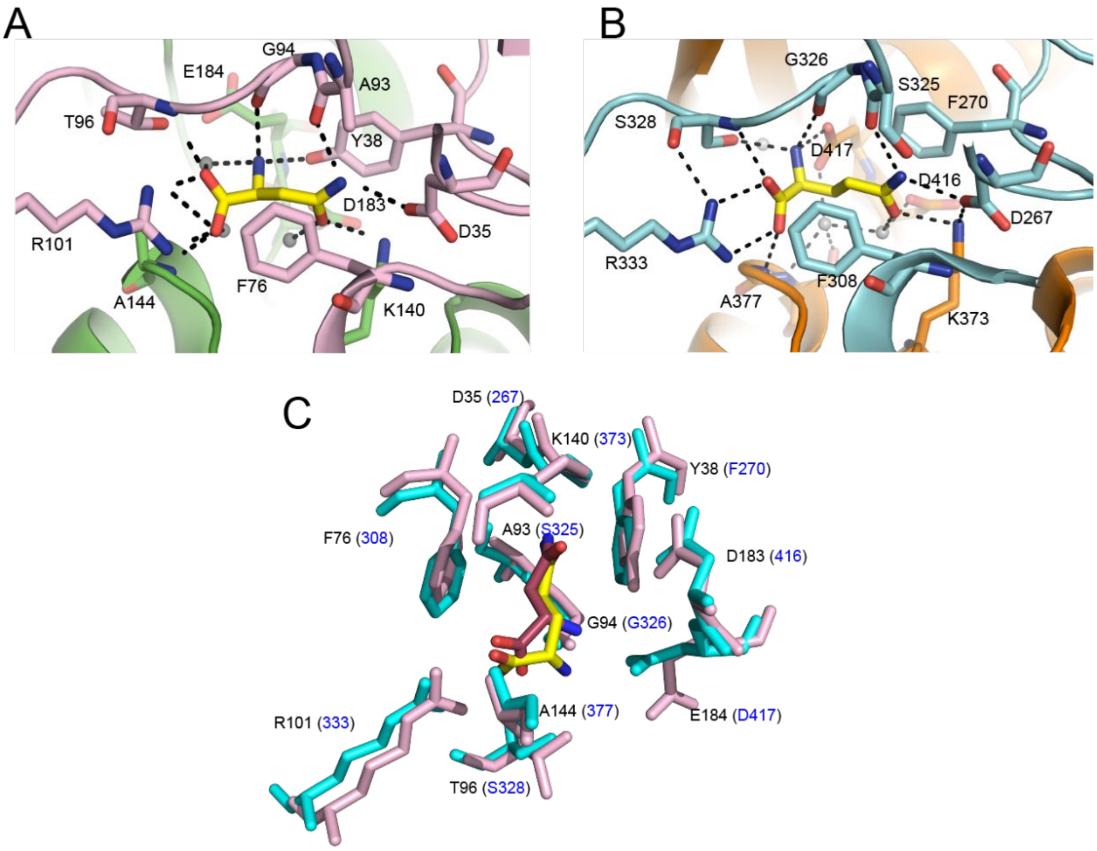

Figure S8. Binding sites of SBD1 and SBD2. (A) Asparagine (yellow) bound to SBD1. In pink residues from the large subdomain are shown; in green the residues and chains from the small subdomain. Residues labelled with numbers have interactions with the substrate. (B) SBD2 with bound glutamine (yellow). In light blue residues from the large subdomain are shown; in orange the residues and chains from the small domain. Residues interacting with glutamine are labelled by number. The dashed lines show polar contacts in the binding sites, which range from 1.8 to 3.6Å. Oriented water molecules are shown by grey balls and these have interactions with residues important for ligand binding. (C) Overlay of the residues in the active site was done by aligning the hinge region, which is the distinguishing features of SBDs. Asparagine is shown in red; glutamine in yellow. Black labels for SBD1 residues; blue labels for SBD2 residues.

Supplementary SI Movie 1

Transitions of WT SBD1 protein 1 from the open to the closed state, regardless of the presence of the ligand in the simulated box.

Supplementary SI Movie 2

The effect of mutation on the opening of the D1 and D2 domains in the E184D mutant.

Supplementary SI Movie 3

Transitions of WT SBD2 protein 1 from the open to the closed state.

Supplementary SI Movie 4

SBD1-Asn recognition and the binding process in the coarse-grained MD simulation

SUPPLEMENTARY REFERENCES

(1) Fulyani, F.; Schuurman-Wolters, G. K.; Slotboom, D.-J.; Poolman, B. Relative Rates of Amino Acid Import via the ABC Transporter GlnPQ Determine the Growth Performance of Lactococcus Lactis. *J. Bacteriol.* **2016**, *198* (3), 477–485.

(2) Fulyani, F.; Schuurman-Wolters, G. K.; Zagar, A. V.; Guskov, A.; Slotboom, D.-J.; Poolman, B. Functional Diversity of Tandem Substrate-Binding Domains in ABC Transporters from Pathogenic Bacteria. *Structure* **2013**, *21* (10), 1879–1888.

(3) Ploetz, E.; Schuurman-Wolters, G. K.; Zijlstra, N.; Jager, A. W.; Griffith, D. A.; Guskov, A.; Gouridis, G.; Poolman, B.; Cordes, T. Structural and Biophysical Characterization of the Tandem Substrate-Binding Domains of the ABC Importer GlnPQ. *Open Biol.* **2021**, *11* (4), 200406.

(4) Gouridis, G.; Schuurman-Wolters, G. K.; Ploetz, E.; Husada, F.; Vietrov, R.; de Boer, M.; Cordes, T.; Poolman, B. Conformational Dynamics in Substrate-Binding Domains Influences Transport in the ABC Importer GlnPQ. *Nat. Struct. Mol. Biol.* **2015**, *22* (1), 57–64.

(5) Cabani, S.; Gianni, P.; Mollica, V.; Lepori, L. Group Contributions to the Thermodynamic Properties of Non-Ionic Organic Solutes in Dilute Aqueous Solution. *Journal of Solution Chemistry*. **1981**, *10*, 563–595.
